# Supplementary material for: The Association between Influenza and Pneumococcal Vaccinations and SARS-Cov-2 Infection: Data from the EPICOVID19 Web-Based Survey
Source: Vaccines (Basel). 2020 Aug 23;8(3):471. doi: 10.3390/vaccines8030471 (PMC7565943; doi:10.3390/vaccines8030471)
Supplement: Supplementary file 1 [file vaccines-08-00471-s001.zip › Supplementary materials--layout.pdf]

Supplementary Figure S1. Response rate to EPICOVID19 x10.000 inhabitants by Italian Regions

Supplementary Figure S2. Age pyramid of the EPICOVID19 and the Italian population (ISTAT, 2019)

Supplementary Figure S3. Education among EPICOVID19 participants and in the Italian population (ISTAT, 2019*) * Population by highest level of education [http://dati.istat.it/Index.aspx?QueryId=26175]

Supplementary Figure S4. Ratio among (total swab performed / total individuals tested at least once) and (total COVID-19 cases / total individuals tested at least once)[21] (higher ratio corresponds to greater regional resources allocated for swab performing)

Supplementary Figure S5. Anti-pneumococcal vaccination percentages among EPICOVID19 participants (left, overall; right, participants with 65 years or more)

Supplementary Table S1. Characteristics of participants according to influenza vaccination during last autumn.

	<65 years			≥65 years		
	**Influenza Vaccination (n=26,809)**	**No influenza Vaccination (n=143,922)**	**p-value**	**Influenza Vaccination (n=15,011)**	**No influenza Vaccination (n=13,086)**	**p-value**
Socio-demographic characteristics						
Sex, males, No. (%)	10793 (40.3)	55589 (38.6)	<0.0001	7770 (51.8)	6015 (46.0)	<0.0001
Age, years, mean±SD	46.8±12.3	43.7±12.0	<0.0001	71.8±5.8	69.7±4.9	<0.0001
Education, No. (%)						
Elementary school or less	111 (0.4)	230 (0.2)	<0.0001	567 (3.8)	392 (3.0)	<0.0001
Middle or High school	8683 (32.4)	57729 (40.1)		6575 (43.8)	6373 (48.7)	
University degree or post-graduate	18015 (67.2)	85963 (59.7)		7869 (52.4)	6321 (48.3)	
Italian area of residence*, No. (%)						
Area 1	15761 (58.9)	84144 (58.6)	0.6470	9079 (60.5)	8192 (62.7)	0.0008
Area 2	3472 (13.0)	18987 (13.0)		1830 (12.2)	1480 (11.3)	
Area 3	5826 (21.8)	31445 (21.9)		3218 (21.5)	2618 (20.0)	
Area 4	1693 (6.3)	9012 (6.3)		869 (5.8)	776 (5.9)	
Smoking status, No. (%) (%)						
Never	16808 (62.7)	83691 (58.2)	<0.0001	7190 (47.9)	6360 (48.6)	<0.0001
Former smoker	6193 (23.1)	30786 (21.4)		6136 (40.9)	4664 (35.6)	
Current	3808 (14.2)	29445 (20.5)		1685 (11.2)	2062 (15.8)	
*Self-reported diseases***						
Lung diseases, No. (%)	2517 (9.4)	6860 (4.8)	<0.0001	1442 (9.6)	637 (4.9)	<0.0001
CVD, No. (%)	2171 (8.1)	5531 (3.8)	<0.0001	4926 (32.8)	2656 (20.3)	<0.0001
Hypertension, No. (%)	4747 (17.7)	14762 (10.3)	<0.0001	8192 (54.6)	5619 (42.9)	<0.0001
Oncological diseases, No. (%)	1156 (4.3)	3033 (2.1)	<0.0001	1522 (10.1)	1008 (7.7)	<0.0001
Depression and/or anxiety, No. (%)	3319 (12.4)	13082 (9.1)	<0.0001	2467 (16.4)	1695 (13.0)	<0.0001
Liver diseases, No. (%)	225 (0.8)	786 (0.6)	<0.0001	263 (1.8)	213 (1.6)	0.4204
Renal diseases, No. (%)	287 (1.1)	846 (0.6)	<0.0001	357 (2.4)	199 (1.5)	<0.0001
Diabetes treated with medications, No. (%)	922 (3.4)	1572 (1.1)	<0.0001	1372 (9.1)	728 (5.6)	<0.0001
Metabolic diseases, No. (%)	3183 (11.9)	8196 (5.7)	<0.0001	5590 (37.2)	3292 (25.2)	<0.0001
Thyroid diseases, No. (%)	2341 (8.7)	9255 (6.4)	<0.0001	1792 (11.9)	1329 (10.2)	<0.0001
Diseases of the immune system, No. (%)	2840 (10.6)	11588 (8.1)	<0.0001	1676 (11.2)	1410 (10.8)	0.2967
Dependency in daily activities, No. (%)	177 (0.7)	216 (0.2)	<0.0001	322 (2.2)	212 (1.6)	0.0013
Self-rated health, No. (%)						
Very bad or Bad	335 (1.3)	914 (0.6)	<0.0001	382 (2.5)	180 (1.4)	<0.0001
Adequate	4455 (16.6)	17317 (12.0)		4704 (31.3)	3273 (25.0)	

	<65 years			≥65 years		
	**Influenza Vaccination (n=26,809)**	**No influenza Vaccination (n=143,922)**	**p-value**	**Influenza Vaccination (n=15,011)**	**No influenza Vaccination (n=13,086)**	**p-value**
Good or Very good	22019 (82.1)	125695 (87.3)		9925 (66.1)	9633 (73.6)	
Anti-pneumococcal vaccination, No. (%)	2511 (9.4)	1206 (0.8)	<0.0001	3099 (20.6)	362 (2.8)	<0.0001
Self-reported symptomatology (02-05.2020), No. (%)						
Fever	2245 (8.4)	13007 (9.0)	0.0005	813 (5.4)	768 (5.9)	0.1004
Headache	7765 (29.0)	44455 (30.9)	<0.0001	1530 (10.2)	1430 (10.9)	0.0453
Myalgia	5539 (20.7)	29400 (20.4)	0.3848	2465 (16.4)	2058 (15.7)	0.1141
Anosmia/dysgeusia	1574 (5.9)	8600 (6.0)	0.5078	457 (3.0)	464 (3.6)	0.0186
Shortness of breath	1536 (5.7)	8405 (5.8)	0.4779	550 (3.7)	417 (3.2)	0.0286
Chest pain	1714 (6.4)	10645 (7.4)	<0.0001	446 (3.0)	430 (3.3)	0.1299
Heart palpitationsGastrointestinal	1784 (6.7)	9645 (6.7)	0.7771	614 (4.1)	520 (4.0)	0.6203
	5043 (18.8)	25523 (17.7)	<0.0001	1481 (9.9)	1229 (9.4)	0.1791
Conjunctivitis	2673 (10.0)	13339 (9.3)	0.0003	1565 (10.4)	1326 (10.1)	0.4205
Pneumonia	198 (0.7)	754 (0.5)	<0.0001	128 (0.9)	83 (0.6)	0.0344
Sore throat/rhinorrhea	9369 (35.0)	49833 (34.6)	0.3088	3151 (21.0)	2606 (19.9)	0.0257
Cough	6089 (22.7)	31628 (22.0)	0.0076	2417 (16.1)	2047 (15.6)	0.2939
No sign or symptoms	9913 (37.0)	53654 (37.3)	0.3452	7981 (53.2)	7187 (54.9)	0.0033
Molecular test for SARS-CoV-2 performed, No. (%)	2043 (7.6)	4621 (3.2)	<0.0001	378 (2.5)	272 (2.1)	0.0145
Contact with confirmed COVID-19 cases, No. (%)	3492 (13.0)	11252 (7.8)	<0.0001	675 (4.5)	606 (4.6)	0.5906

*Area 1: Piedmont, Lombardy, Emilia Romagna, Liguria, Marche, Aosta Valley; Area 2: Tuscany, Trentino Alto Adige, Abruzzo, Apulia; Area 3: Veneto, Lazio, Friuli Venezia Giulia, Molise, and Campania; Area 4: Sicily, Sardinia, Umbria, Calabria, Basilicata. **: defined considering both self-reported diseases and medications used; SD: Standard Deviation

Supplementary Table S2. Characteristics of participants according to anti-pneumococcal vaccination in the last 12 months

	<65 years			≥65 years		
	**Anti-pneumococcal Vaccination (n=3717)**	**No Anti-pneumococcal Vaccination (n=167014)**	**p-value**	**Anti-pneumococcal Vaccination (n=3461)**	**No Anti-pneumococcal Vaccination (n=24636)**	**p-value**
Socio-demographic characteristics						
Sex, males, No. (%)	1668 (44.9)	64714 (38.8)	<0.0001	1720 (49.7)	12065 (49.0)	0.4253
Age, years, mean±SD	41.4±14.4	44.3±12.0	<0.0001	70.4±5.2	70.8±5.6	<0.0001
Education, No. (%)						
Elementary school or less	55 (1.5)	286 (0.2)	<0.0001	55 (1.6)	904 (3.7)	<0.0001
Middle or High school	1530 (41.2)	64882 (38.9)		1446 (41.8)	11502 (46.7)	
University degree or post-graduate	2132 (57.4)	101846 (61.0)		1960 (56.6)	12230 (49.6)	
Italian area of residence*, No. (%)						
Area 1	2020 (54.5)	97885 (58.7)	<0.0001	1851 (53.5)	15420 (62.7)	<0.0001
Area 2	574 (15.5)	21885 (13.1)		437 (12.6)	2873 (11.7)	
Area 3	840 (22.7)	36431 (21.9)		982 (28.4)	4854 (19.7)	
Area 4	270 (7.3)	10435 (6.3)		187 (5.4)	1458 (5.9)	
Smoking status, No. (%)						
Never	2264 (60.9)	98235 (58.8)	0.0244	1553 (44.9)	11997 (48.7)	<0.0001
Former smoker	783 (21.1)	36196 (21.7)		1473 (42.6)	9327 (37.9)	
Current	670 (18.0)	32583 (19.5)		435 (12.6)	3312 (13.5)	
*Self-reported diseases***						
Lung diseases, No. (%)	498 (13.4)	8879 (5.3)	<0.0001	541 (15.6)	1538 (6.2)	<0.0001
CVD, No. (%)	329 (8.9)	7373 (4.4)	<0.0001	1079 (31.2)	6503 (26.4)	<0.0001
Hypertension, No. (%)	538 (14.5)	18971 (11.4)	<0.0001	1824 (52.7)	11987 (48.7)	<0.0001
Oncological diseases, No. (%)	180 (4.8)	4009 (2.4)	<0.0001	388 (11.2)	2142 (8.7)	<0.0001
Depression and/or anxiety, No. (%)	486 (13.1)	15915 (9.5)	<0.0001	546 (15.8)	3616 (14.7)	0.0886
Liver diseases, No. (%)	45 (1.2)	966 (0.6)	<0.0001	61 (1.8)	415 (1.7)	0.7393
Renal diseases, No. (%)	71 (1.9)	1062 (0.6)	<0.0001	90 (2.6)	466 (1.9)	0.0050
Diabetes treated with medications, No. (%)	116 (3.1)	2378 (1.4)	<0.0001	312 (9.0)	1788 (7.3)	0.0002
Metabolic diseases, No. (%)	382 (10.3)	10997 (6.6)	<0.0001	1274 (36.8)	7608 (30.9)	<0.0001
Thyroid diseases, No. (%)	263 (7.1)	11333 (6.8)	0.4871	420 (12.1)	2701 (11.0)	0.0400
Diseases of the immune system, No. (%)	452 (12.2)	13976 (8.4)	<0.0001	419 (12.1)	2667 (10.8)	0.0241
Dependency in daily activities, No. (%)	65 (1.8)	328 (0.2)	<0.0001	51 (1.5)	483 (2.0)	0.0494
Self-rated health, No. (%)						
Very bad or Bad	74 (2.0)	1175 (0.7)	<0.0001	96 (2.8)	466 (1.9)	<0.0001
Adequate	720 (19.4)	21048 (12.6)		1087 (31.4)	6890 (28.0)	

	<65 years			≥65 years		
	**Anti-pneumococcal Vaccination (n=3717)**	**No Anti-pneumococcal Vaccination (n=167014)**	**p-value**	**Anti-pneumococcal Vaccination (n=3461)**	**No Anti-pneumococcal Vaccination (n=24636)**	**p-value**
Good or Very good	2923 (78.6)	144791 (86.7)		2278 (65.8)	17280 (69.1)	
Flu shot during last autumn, No. (%)	2511 (67.6)	24298 (14.6)	<0.0001	3099 (89.5)	11912 (48.4)	<0.0001
Self-reported symptomatology (02-05.2020), No. (%)						
Fever	328 (8.8)	14924 (8.9)	0.8137	196 (5.7)	1385 (5.6)	0.9215
Headache	1110 (29.9)	51110 (30.6)	0.3332	363 (10.5)	2597 (10.5)	0.9240
Myalgia	708 (19.1)	34231 (20.5)	0.0304	581 (16.8)	3942 (16.0)	0.2387
Anosmia/dysgeusia	194 (5.2)	9980 (6.0)	0.0541	113 (3.3)	808 (3.3)	0.9335
Shortness of breath	257 (6.9)	9684 (5.8)	0.0041	159 (4.6)	808 (3.3)	<0.0001
Chest pain	269 (7.2)	12090 (7.2)	0.9965	110 (3.2)	766 (3.1)	0.8269
Heart palpitations	252 (6.8)	11177 (6.7)	0.8330	165 (4.8)	969 (3.9)	0.0195
Gastrointestinal	707 (19.0)	29859 (17.9)	0.0723	338 (9.8)	2372 (9.6)	0.7971
Conjunctivitis	349 (9.4)	15663 (9.4)	0.9818	381 (11.0)	2510 (10.2)	0.1370
Pneumonia	27 (0.7)	925 (0.6)	0.1623	42 (1.2)	169 (0.7)	0.0008
Sore throat/rhinorrhea	1276 (34.3)	57926 (34.7)	0.6533	748 (21.6)	5009 (20.3)	0.0806
Cough	847 (22.8)	36870 (22.1)	0.3013	613 (17.7)	3851 (15.6)	0.0017
No sign or symptoms, No. (%)	1408 (37.9)	62159 (37.2)	0.4088	1780 (51.4)	13388 (54.3)	0.0013
Molecular test for SARS-CoV-2 performed, No. (%)	246 (6.6)	6418 (3.8)	<0.0001	102 (3.0)	548 (2.2)	0.0081
Contact with confirmed COVID-19 cases, No. (%)	415 (11.2)	14329 (8.6)	<0.0001	156 (4.5)	1125 (4.6)	0.8759

* Area 1: Piedmont, Lombardy, Emilia Romagna, Liguria, Marche, Aosta Valley; Area 2: Tuscany, Trentino Alto Adige, Abruzzo, Apulia; Area 3: Veneto, Lazio, Friuli Venezia Giulia, Molise, and Campania; Area 4: Sicily, Sardinia, Umbria, Calabria, Basilicata. **: defined considering both self-reported diseases and medications used; SD: Standard Deviation
