# Supplementary figures and images for: The Association between Influenza and Pneumococcal Vaccinations and SARS-Cov-2 Infection: Data from the EPICOVID19 Web-Based Survey
Source: Vaccines (Basel). 2020 Aug 23;8(3):471. doi: 10.3390/vaccines8030471 (PMC7565943; doi:10.3390/vaccines8030471)

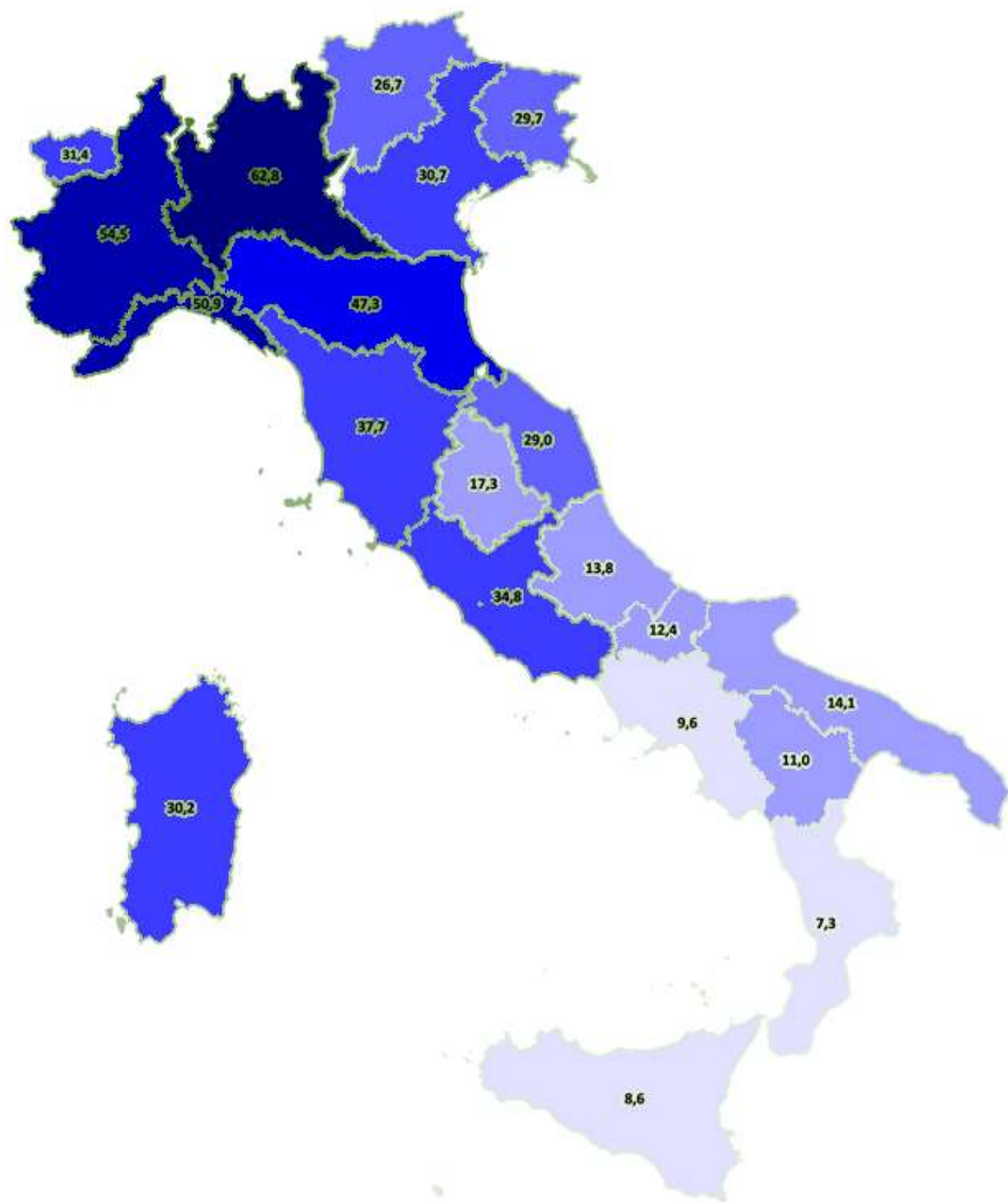

Supplement: Supplementary file 1 [file vaccines-08-00471-s001.zip › Figure S1.pdf]

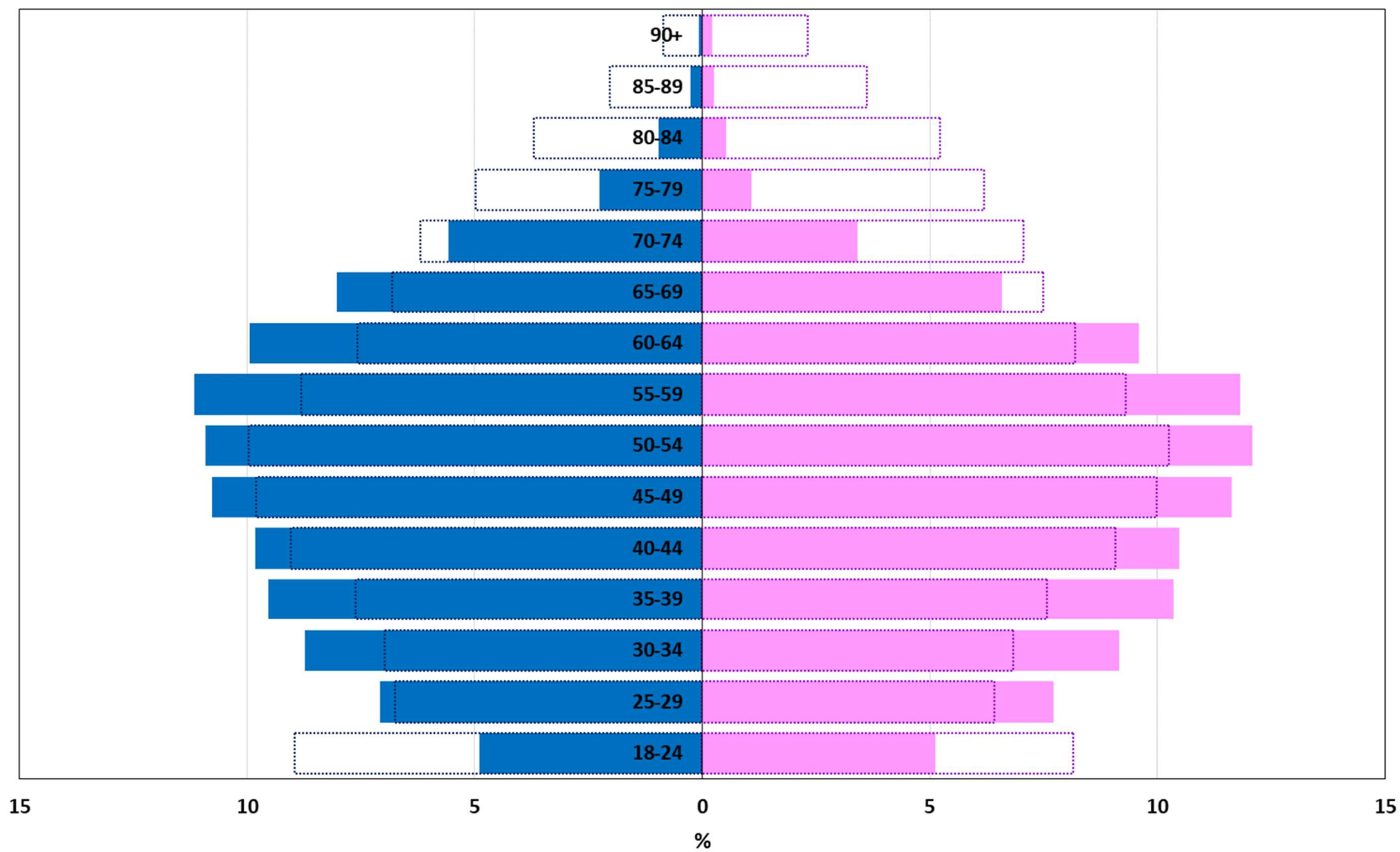

■ Females, ISTAT 2019  
 ■ Males, ISTAT 2019  
 ■ Males, EPICOV19  
 ■ Females, EPICOV19

Supplement: Supplementary file 1 [file vaccines-08-00471-s001.zip › Figure S2.pdf]

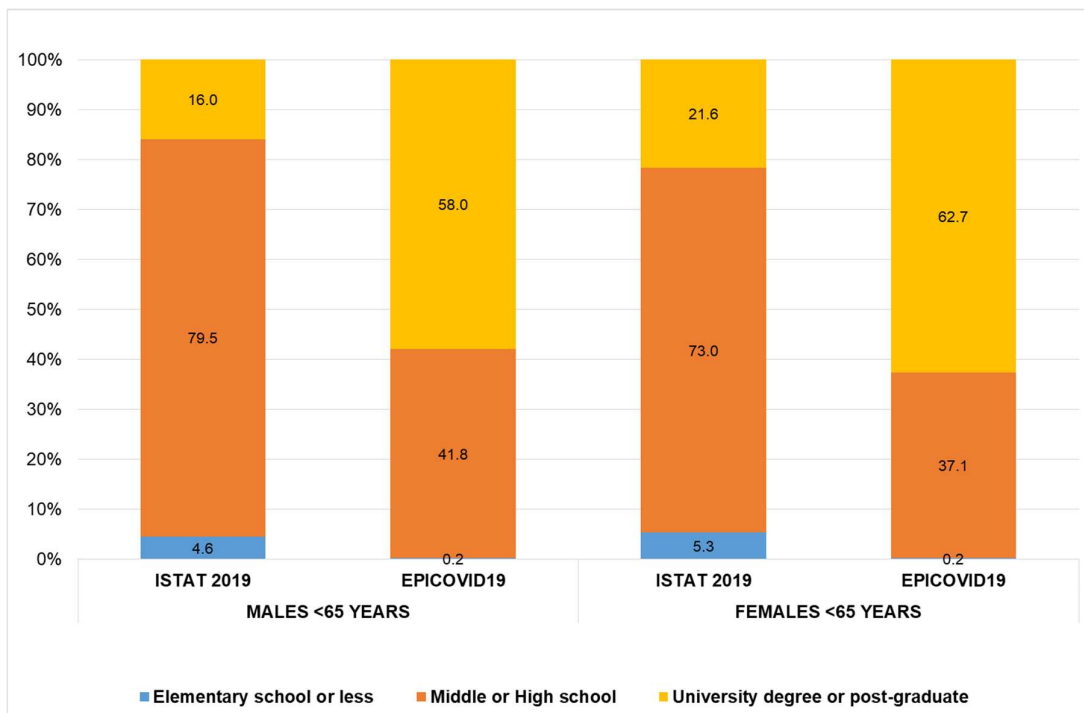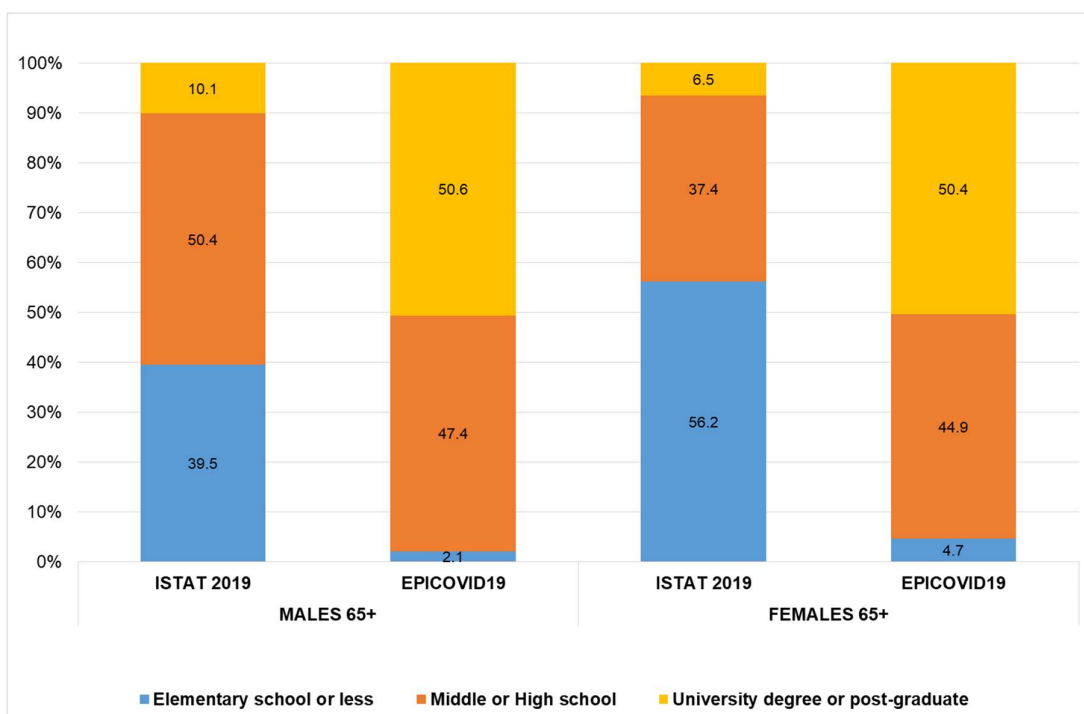

Supplement: Supplementary file 1 [file vaccines-08-00471-s001.zip › Figure S3.pdf]

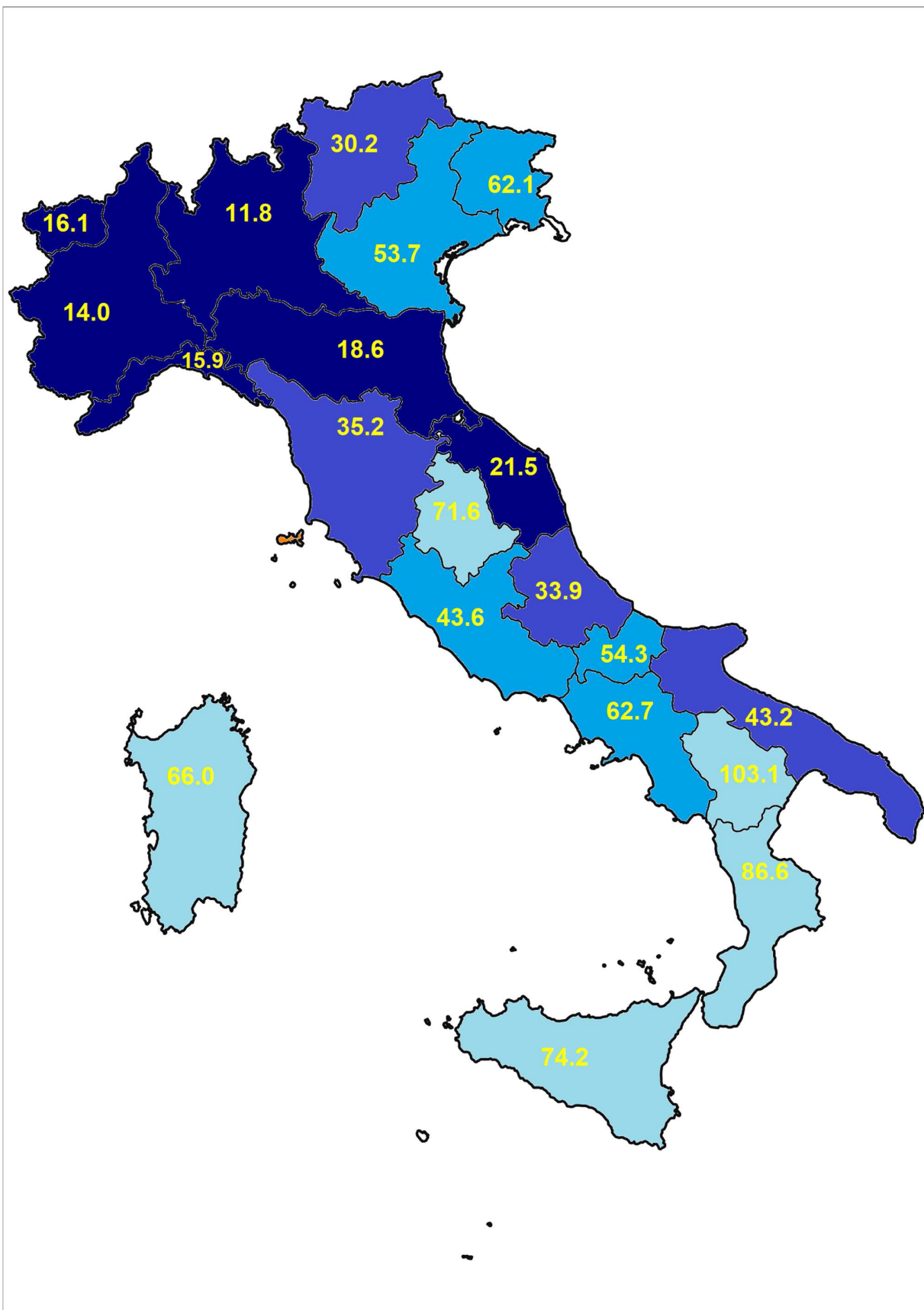

Supplement: Supplementary file 1 [file vaccines-08-00471-s001.zip › Figure S4.pdf]

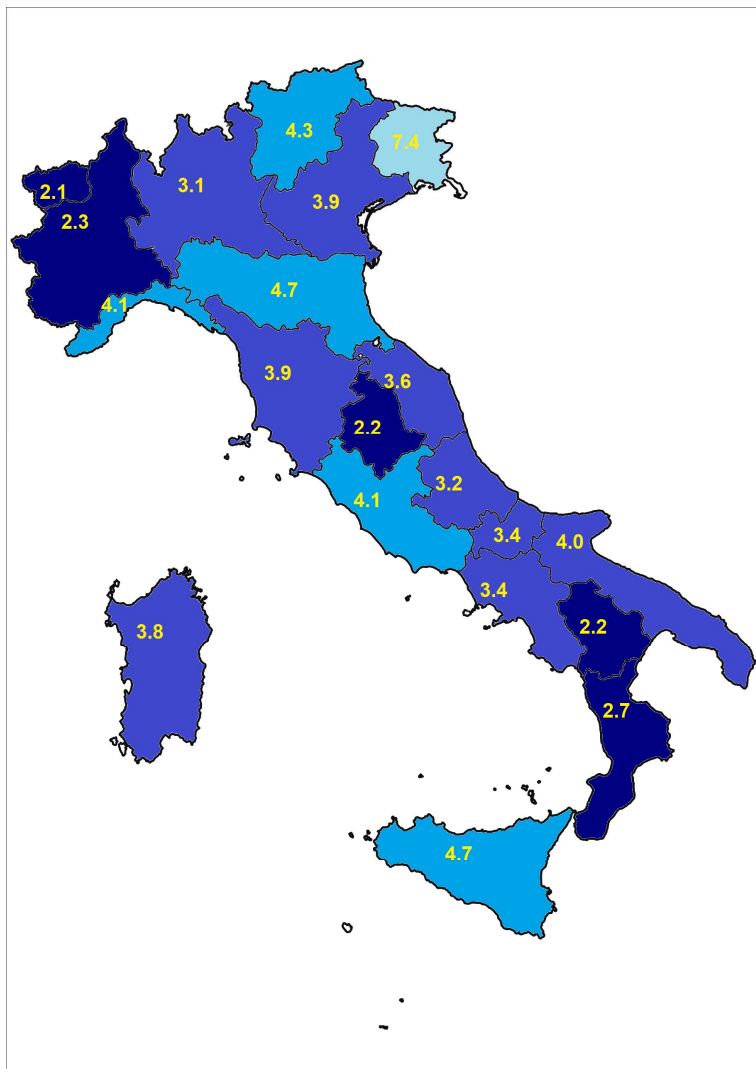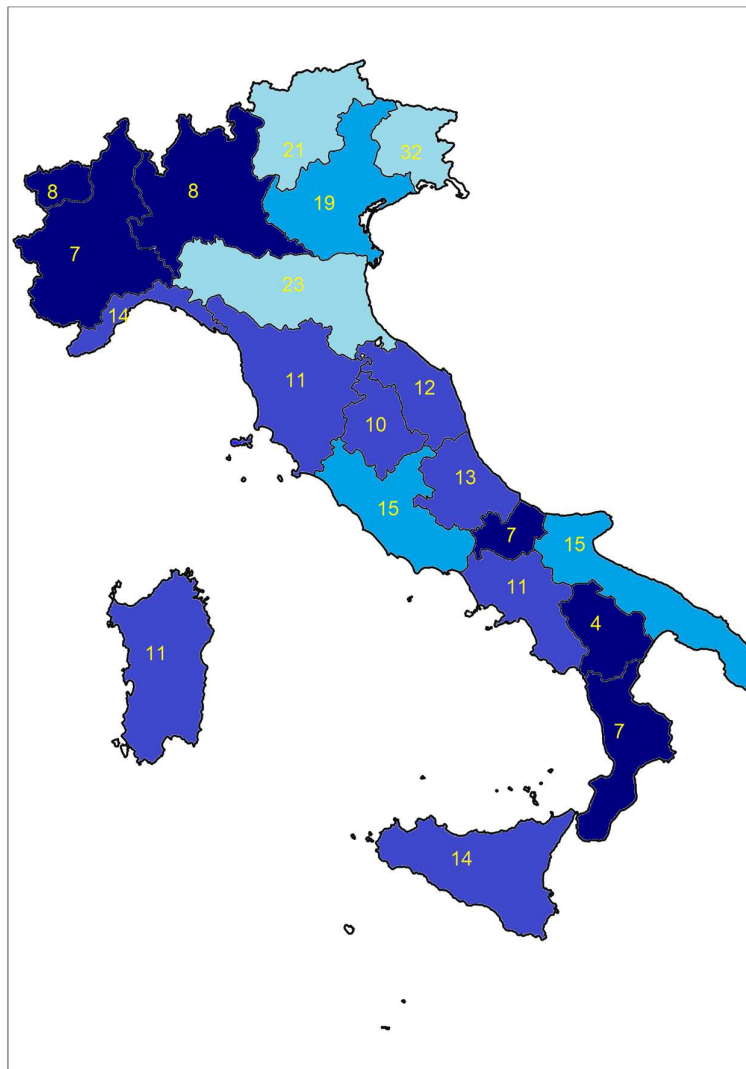

Supplement: Supplementary file 1 [file vaccines-08-00471-s001.zip › Figure S5.pdf]
